# Supplementary material for: Caregiver experience and perceived acceptability of a novel near point-of-care early infant HIV diagnostic test among caregivers enrolled in the PMTCT program, Myanmar: A qualitative study
Source: PLoS One. 2020 Oct 30;15(10):e0241245. doi: 10.1371/journal.pone.0241245 (PMC7598472; doi:10.1371/journal.pone.0241245)
Supplement: S1 File — (DOCX) [file pone.0241245.s001.docx]

**“Accelerating HIV testing and ART initiation among infants (AAMI)” Study Group**

- Burnet Institute, Melbourne, Australia
  - Professor Stanley Luchters, Principal Investigator
  - Professor Suzanne Crowe, Co-investigator
  - Associate Professor Mark Stoové, Co-investigator
  - Associate Professor David Anderson, Co-investigator
  - Dr Claire Nightingale, Co-investigator
  - Mr Paul Agius, Co-investigator
  - Ms Yasmin Mohamed, Research Coordinator
- Burnet Institute, Yangon, Myanmar
  - Dr Hla Htay, lead Principal Investigator Myanmar
  - Dr Win Lei Yee, Research Coordinator Myanmar
- The Kirby Institute for infection and immunity in society, University of New South Wales, Sydney, Australia
  - Dr Angela Kelly-Hanku, co-Principal Investigator Papua New Guinea
  - Associate Professor Andrew Vallely, co-Principal Investigator Papua New Guinea
  - Dr Steven Badman, Co-investigator
- Mt Hagen General Hospital, Mt Hagen, Papua New Guinea
  - Dr Zure Kombati, Co-investigator Papua New Guinea
- National AIDS/STD Control Program, Ministry of Health and Sports, Myanmar
  - Dr Tin Maung Zaw, Co-investigator Myanmar
- National Center for STD Control, Nanjing, China
  - Professor Xiang-Sheng Chen, Co-investigator
- National Health Laboratory, Yangon, Myanmar
  - Professor Htay Htay Tin, co-Principal Investigator Myanmar
  - Dr Win Thein, co-Principal Investigator Myanmar
  - Dr Latt Latt Kyaw, Co-investigator Myanmar
- Papua New Guinea Institute of Medical Research, Goroka, Papua New Guinea
  - Dr Janet Gare, Research Coordinator Papua New Guinea
  - Ms Selina Silim, Research Officer Papua New Guinea
