# Supplementary material for: Caregiver experience and perceived acceptability of a novel near point-of-care early infant HIV diagnostic test among caregivers enrolled in the PMTCT program, Myanmar: A qualitative study
Source: PLoS One. 2020 Oct 30;15(10):e0241245. doi: 10.1371/journal.pone.0241245 (PMC7598472; doi:10.1371/journal.pone.0241245)
Supplement: S2 File — (DOCX) [file pone.0241245.s002.docx]

# AAMI Study

## Interview guide for caregivers bringing their child for EID results

**Materials and supplies**

- Consent forms (one copy for participant, one copy for the team)
- Question guide for interviewer
- One digital recording device
- Spare Battery for recording device
- Notebook for note-taking and pens
- Refreshments

**To be completed for each interview:**

| Name of interviewer: |  | Date (dd/mm/yyyy): |  |
| --- | --- | --- | --- |
| Start time: |  | End time: |  |
|  | | | |
| Age of caregiver: |  | Facility Number: | **\|___\|___\|** |
| Participant ID: | **\|___\|___\|___\|___\| - \|___\|** | Has written informed consent been obtained? | Yes / No |

| **Theme / topic** | **Question** | **Probe** |
| --- | --- | --- |
| Opening questions | 1. Can you tell me a little bit about yourself? | *Where are you from?*  *Are you married?*  *Who do you live with?*  *How old are you?*  *How many children do you have?* |
|  | 1. Can you tell me a bit about your baby? | *How old is your baby?*  *What is their name?*  *How is your baby sleeping?*  *How is your baby feeding? What are you giving them to eat/drink?*  *How are you coping with being a new mum?*  *What supports do you have at home?* |
| Perceptions of the quality of services | 1. Can you tell me about your involvement in the PMTCT program? | *What is the care you and your baby receive here like?*  *How do you find the health care staff? How do they treat you and your baby?*  *What do you expect the clinic to do for you?*  *What are the best things about this clinic?*  *What are the worst things about this clinic?*  *How could this clinic be improved?* |
| Barriers to accessing laboratory tests for their child | 1. Can you tell me about the birth of your baby? | *How did everything go?*  *Did you have a supervised delivery?*  *Did you deliver at a health facility? What were some of the reasons for this?*  *If you delivered at the health facility, what was it that made you want to deliver here?* |
|  | 1. Some women don’t come back to the clinic after the birth of their baby. What was it that made you want to come back to the clinic? | *What makes it difficult for you to come to the clinic?*  *What makes it easier for you to come back to the clinic?*  *Did you feel like you had to come back to the clinic, or was it your choice to come back?*  *What do you hear about other women coming back to the clinic? What makes it hard for them to come back?* |
|  | 1. Can you tell me about how you get to the clinic? | *How easy is it for you to travel to the clinic?*  *How far is it for you?*  *What sort of support do you have to help you get to the clinic? Who came with you?*  *If you have other kids, who looks after them?* |
|  | 1. Can you tell me about how you get an appointment at clinic? | *Were there times when the appointments didn’t work for you?*  *How easy was it for you to attend the clinic on another day?*  *How often did you come to the clinic and it was too busy for you to get seen?*  *How long do you normally have to wait to be seen at the clinic?* |
| Experiences of having an infant enrolled in a study and an understanding of the information, counselling and consent process used in the study process | 1. Can you tell me about when you first found out about the study? | *What were you told about the study?*  *What were your thoughts about the study?*  *Did you have any worries or concerns?*  *Were all your concerns addressed?*  *Were all your questions about the study answered?*  *Do you feel you understood everything that was happening as part of the study?* |
|  | 1. Why did you decide to take part in the study? | *Did you discuss the study with anyone else before you decided to take part? Why/why not?*  *If yes, whom did you discuss the study with?* |
|  | 1. Can you tell me what it was like once you decided to take part in the study? | *Did you tell anyone about taking part in the study? Why/why not?*  *If yes, whom did you tell?*  *Can you tell me about what changed for you once you started the study?*  *What impact has the study had on you or your family?*  *Were you worried about what the study team were going to do? Why/why not?*  *Do you have any regrets about taking part in the study?* |
|  | 1. What did you think about the new HIV test that is being used in the study? | *Had you heard of this test?*  *What were you told about this test?*  *What did you think about this test when you were told we could give you results on the same day?* |
| *I am now going to ask you some questions about HIV. Some of these questions may be upsetting to you. You do not have to answer any questions you don’t want to and we can stop the interview at any time.* | | |
| Knowledge about infant diagnosis of HIV and how they relate to the need for ART | 1. When you found out you were positive or first came to the PMTCT clinic, what did the doctors or nurses tell you about testing your baby for HIV? | *Did they tell you how long you would have to wait?*  *What did they tell you about how to care for your baby while waiting for the result?*  *Did they talk to you about what would happen if your baby did have HIV?*  *Did they tell you about medication your baby would need to take just after he/she was born?* |
| ART prophylaxis | 1. Can you tell me about the medication your baby was given after he/she was born to prevent HIV? | *How soon after your baby was born were you given this medication?*  *What did the doctors or nurses tell you about the medication?*  *What was it like getting your baby to take this medication?*  *What were some of the challenges?*  *What supports did you have?*  *Did you have any worries or concerns about this?* |
| Experiences of having their child undergo EID | 1. When it came to the day when your baby had the HIV test, what was it like? | *Tell me about the day.*  *Did you have any worries or concerns?*  *Did you come on the day they asked you to come?*  *How long did you have to wait to see the doctor or nurse?*  *What did you do while you waited?* |
|  | 1. What were some of the reasons that you brought your baby in for testing? | *What made it easier to bring your baby to the clinic?*  *What made it more difficult?* |
|  | 1. Can you tell me what it was like waiting for your baby’s test result? | *How long did you have to wait for the result?*  *What did you do while you waited?* |
|  | 1. Can you tell me about when the doctor or nurse told you your baby’s result? | *How did they tell you?*  *What did they tell you?*  *What instructions did they give you?*  *What was it like for you to get the result? How did you feel?*  *What were your worries or concerns?*  *Did you tell anyone? Who did you tell?* |
|  | 1. **If the result was positive**, what did the doctors or nurses tell you about your child having HIV? | *What did they tell you about treatment for HIV?*  *Was your baby put on treatment? How long after the result was known?*  *How long did you have to wait for the treatment? Did you come back for another appointment?*  *What was it like starting your baby on treatment?*  *What were some of the challenges?*  *Are you still able to get enough treatment for your baby?*  *How did knowing your baby had HIV change the way you care for them?* |
|  | 1. **If the result was negative**, what did the doctors or nurses tell you about the test result? | *What were you told about further testing?*  *How did knowing your baby didn’t have HIV change the way you care for them?*  *What do you think about being told your baby is negative and being told to breastfeed for 6 months?* |
| *The HIV test your baby had takes about one and a half hours to complete. The standard laboratory test usually takes around one or two months for the results to come back.* | | |
| Acceptability of same day results | 1. What was it like getting your baby’s HIV test result on the same day? | *Do you think you had time to adjust to what your baby’s result might be?*  *What do you see as the benefits or problems getting the test results the same day that the baby is tested?*  *If you have other children, how was the test used in the study different from how your other kids were tested?*  *Can you tell me about the pros and cons of the different approaches?*  *If you had a choice, would you prefer your baby to have the test that takes an hour and a half, or the test that takes a few months to come back?*  *What are some of the reasons for this?* |
| Validity of same day tests (as opposed to tests done in formal laboratories) | 1. Can you tell me about the way babies are usually tested for HIV? | *Who tests the blood sample?*  *Where are they tested?*  *How long does it take to get the results?* |
|  | 1. Normally at the clinic, blood samples are sent the lab to be tested for HIV. What do you think about having the test done at the clinic compared with the one sent to the lab? | *Does it matter that the test was done here at clinic rather than in the lab?*  *Do you think there’s a difference in the reliability between the test done here and in the lab?*  *How reliable do you think the results of the test done for the study are?*  *Do you believe the test results?*  *Have you questioned them? Why did you question them?* |
| Closing question | 1. What would you tell other HIV positive mums /parents about this test and its benefits or difficulties? | *What are the benefits of this test?*  *What are some of the difficulties of this test?*  *Do you want the HIV test to be done differently in the clinic? If so, what changes would you make?* |
