# Supplementary material for: Caregiver experience and perceived acceptability of a novel near point-of-care early infant HIV diagnostic test among caregivers enrolled in the PMTCT program, Myanmar: A qualitative study
Source: PLoS One. 2020 Oct 30;15(10):e0241245. doi: 10.1371/journal.pone.0241245 (PMC7598472; doi:10.1371/journal.pone.0241245)
Supplement: S3 File — (DOCX) [file pone.0241245.s003.docx]

# AAMI Study

## အုပ်ထိန်းသူ/ပြုစုစောင့်ရှောက်သူများအတွက် မေးခွန်းမေးမြန်းရန် လမ်းညွှန်ချက်

**လိုအပ်သောပစ္စည်းများ**

- သဘောတူခွင့်ပြုချက်တောင်းခံလွှာများ (ပါဝင်သူအတွက် တစ်စောင်၊ သုတေသနအဖွဲ့မှ သိမ်းထားရန် တစ်စောင်)
- မေးခွန်းမေးမြန်းသူအတွက် မေးခွန်းလမ်းညွှန်ချက်
- အသံသွင်းစက် တစ်ခု
- အသံသွင်းစက်အတွက် ဘက်ထရီ အပို တစ်ခု
- မှတ်စုရေးရန် စာအုပ်နှင့်ဘောပင်များ
- ပါဝင်သူအတွက် အစားအသောက်ဖြင့်ဧည့်ခံရန်

**မေးခွန်းမေးမြန်းခြင်းတစ်ခုစီအတွက် ဖြည့်ရန် -**

| မေးခွန်းမေးမြန်းသူ အမည် |  | နေ့စွဲ  (ရက်/လ/နှစ်) |  |
| --- | --- | --- | --- |
| စတင်ချိန် |  | ပြီးဆုံးချိန် |  |
|  | | | |
| အုပ်ထိန်းသူအသက်: |  | ကျန်းမာရေးဌာန | **\|___\|___\|** |
| ပါဝင်သူ ကုဒ်အမှတ်: | **\|___\|___\|___\|___\| - \|___\|** | စာဖြင့်ရေးသားထားသော သဘောတူခွင့်ပြုကြောင်း ရရှိသည်။ | ရသည်/ မရပါ |

| ခေါင်းစဥ် | မေးခွန်း | ထပ်မံမေးရန် |
| --- | --- | --- |
| အဖွင့်မေးခွန်းများ | ၁။ သင့်အကြောင်း ကျွန်ုပ်ကို အနည်းငယ်ပြောပြပါ။ | သင်ဘယ်မှာနေတာလဲ။  အိမ်ထောင်ရှိပါသလား။  သင်ဘယ်သူနဲ့နေပါသလဲ။  အသက်ဘယ်နှစ် နှစ်ရှိပါပြီလဲ။  ကလေးဘယ်နှစ်ယောက်ရှိပါသ လဲ။ |
|  | ၂၊ သင့်ကလေးအကြောင်း အနည်းငယ်ပြောပြပါ။ | သင့်ကလေးအသက်ဘယ်လောက် လဲ။  နာမည်ရှိပါသလား။  သင့်ကလေး ကောင်းစွာအိပ်စက် ပါသလား။  ကောင်းစွာစားပါသလား။  အစားအသောက်အတွက် ကလေး ကို ဘာတွေကျွေးပါသလဲ။  အမေတစ်ယောက်ဖြစ်လာရတဲ့ အပေါ် သင် မည်သို့ခံစားရပါ သလဲ။  အိမ်တွင်မည်သည့်အထောက်အပံ့ များသင်ရပါသလဲ။ |
| ဝန်ဆောင်မှုအရည်အသွေး အပေါ် ထင်မြင်ယူဆချက် များ | ၃။ PMTCT အစီအစဥ်တွင် ပါဝင်ခဲ့ပုံအကြောင်းကို ပြောြပ ပါ။ | *သင် နှင့် သင့်ကလေး မည်သည့် ကျန်းမာရေးစောင့်ရှောက်မှု များ ရရှိနေပါသလဲ။*  *ကျန်းမာရေးဝန်ထမ်းများ အပေါ် မည်သို့ထင်ပါသလဲ။ သင်နှင့် သင့် ကလေး အပေါ် မည်သို့ ဆက်ဆံ ပါသလဲ။*  *မည်သည့်အရာများကိုဆေးခန်းမှ ပြုလုပ်ပေးရန် သင်မျှော်လင့်ပါ သလဲ။*  *ဒီဆေးခန်းမှာ ဘယ်အရာတွေကို သင်အကြိုက်ဆုံးလဲ။*  *ဒီဆေးခန်းမှာ သင်မကြိုက်ဆုံး အရာတွေက ဘာလဲ။*  *ဒီဆေးခန်းပိုကောင်းလာအောင် ဘယ်လိုလုပ်သင့်သလဲ။* |
| ကလေးအတွက် သွေးစစ် ဆေးမှုများပြုလုပ်ရန် အခက် အခဲအတားအဆီးများ | ၄။ သင့်ကလေးမွေးဖွားခဲ့သည့် အကြောင်းကို ပြောပြပါ။ | *အခြေအနေမည်သို့ရှိခဲ့ပါသလဲ။*  *တစ်စုံတစ်ဦး၏ကြီးကြပ်မှု ဖြင့် မွေးဖွားခဲ့ပါသလာ။*  *ကျန်းမာရေးဌာနတွင် မွေးဖွားခဲ့ ပါသလား။ မည်သည့်အကြောင်း ရင်းများကြောင့် ကျန်းမာရေး ဌာန တွင် မွေးဖွားခဲပါသလဲ။*  *အဘယ်ကြောင့် ထို ကျန်းမာရေး ဌာနတွင် မွေးဖွားခဲ့ ပါသလဲ။* |
|  | ၅။ ကလေးမွေးဖွားပြီးနောက် တွင် အချို့အမျိုးသမီးများသည် ဆေးခန်းသို့ပြန်မလာကြပါ။ မည်သည့်အကြောင်းကြောင့် သင့်ဆေးခန်းသို့ပြန်လာဖြစ်ခဲ့ ပါသလဲ။ | *ဆေးခန်းသို့ပြန်လာရန် မည်သည့် အခက်အခဲများရှိပါသလဲ။*  *ဆေးခန်းသို့ပြန်လာရန် မည်သည့် အရာများက လွယ်ကူစေခဲ့ပါ သလဲ။*  *ဆေးခန်းသို့ မဖြစ်မနေပြန်လာ ရမည်ဟု သင်ထင်ပါသလား (သို့) ဆေးခန်းသို့ပြန်လာသည်မှာ သင့် ရွေးချယ်မှုလား။*  *ဆေးခန်းသို့ပြန်လာပြသော အခြား အမျိုးသမီးများ အကြောင်း သင်ဘာကြားမိပါသလဲ။ သူတို့ ပြန်လာရန် မည်သည့်အခက် အခဲများ ရှိပါသလဲ။* |
|  | ၆။ ဆေးခန်းသို့မည်သို့ လာရောက် သည်ကို ပြောပြပါ။ | *ဆေးခန်းလာရာတွင် သင့်အတွက် မည်မျှ လွယ်ကူပါသလဲ။*  *ဆေးခန်းသည် သင်နှင့်မည်မျှ ကွာဝေးပါသလဲ။*  *ဆေးခန်းပြန်လာပြရာတွင် မည် သည့်အကူအညီများ သင်ရရှိ ပါသလဲ။ သင်နှင့်အတူ မည်သူ လိုက်လာပါသလဲ။*  *သင့်တွင်အခြားကလေးများ ရှိပါက သူတို့ကို မည်သူက စောင့်ရှောက် ထားပါသလဲ။* |
|  | ၇။ ဆေးခန်းရက်ချိန်း မည်သို့ ယူသည်ကို ပြောပြနိုင်ပါ သလား။ | *ဆေးခန်းရက်ချိန်းအဆင်မပြေ ခဲ့သည်များ ရှိပါသလား။*  *နောက်တစ်ရက် ဆေးခန်းသို့ ပြန်လာရန် သင်မည်မျှအဆင် ပြေပါသလဲ။*  *သင်ဆေးခန်းလာပြချိန် တွင် ဝန်ထမ်းများအလုပ်ရှုပ် နေသဖြင့် သင်မပြ လိုက်ရသောကြောင့် ပြန်လာရသော အကြိမ်အရေ အတွက် မည်မျှ ရှိပါသလဲ။*  *ဆေးခန်းတွင် ပုံမှန်အားဖြင့် သင် အချိန်မည်မျှစောင့်ရပါသလဲ။* |
| ကလေးအတွက်သုတေသနတွင် ပါဝင်သော အတွေ့အကြုံ နှင့် သုတေ သနအကြောင်း၊ နှစ်သိမ့် ဆွေးနွေးခြင်း နှင့် သဘောတူခွင့်ပြုလွှာတောင်းခံ ခြင်း တို့အပေါ် နားလည်မှု | ၈။ သုတေသနအကြောင်း သင်စတင် သိသည့်အချိန် အကြောင်း ပြောပြပါ။ | *သင့်အား သုတေသနအကြောင်း ဘာတွေပြောပြပါသလဲ။*  *သုတေသနနှင့်ပတ်သက်၍ သင်ဘာတွေ စဥ်းထားခဲ့ပါသလဲ။*  *သင့် စိုးရိမ်ပူပန်သည့်အရာများ ရှိခဲ့ ပါသလား။*  *သင်စိတ်ပူနေသည့်အရာများ ကို ဖြေရှင်းပေးခဲ့ပါသလား။*  *သုတေသနနှင့်ပတ်သက်၍ သင် သိလိုသည်များကို မေးမြန်းရာတွင် ဖြေကြား ပေးခဲ့ပါသလား။*  *သုတေသနတွင် ပြုလုပ်သည့် အရာမှန်သမျှကို သင်နားလည် သည်ဟု ထင်ပါသလား။* |
|  | ၉။ သုတေသနတွင်ပါဝင်ရန် သင်ဘာကြောင့်ဆုံးဖြတ်ခဲ့ ပါသလဲ။ | *ပါဝင်ရန်မဆုံးဖြတ်မီ အခြားသူ တစ်ဦးနှင့် သင်ဆွေးနွေးခဲ့ပါသ လား။ ဘာကြောင့်ဆွေးနွေးခဲ့ပါ သလဲ/ဘာကြောင့်မဆွေးနွေးခဲ့ ပါသလဲ။*  *ဆွေးနွေးခဲ့ပါက မည်သူနှင့် ဆွေးနွေး ခဲ့ပါသလဲ။* |
|  | ၁၀။ သုတေသနတွင်ပါဝင် ရန် သင်ဆုံးဖြတ်ပြီးသည့် အခါ မည်ကဲ့သို့အခြေအနေ ရှိသည် ကို ပြောပြပါ။ | *သုတေသနတွင်သင်ပါဝင်ထား ကြောင်းကို တစ်စုံတစ်ဦးအား ပြောပြခဲ့ပါသလား။ ဘာကြောင့် ပြောပြခဲ့ပါလဲ/ ဘာကြောင့် မပြောပြခဲ့ပါလဲ။*  *ပြောပြခဲ့ပါက မည်သူ့ကို ပြောပြ ခဲ့ပါသလဲ။*  *သုတေသနတွင်ပါဝင်ပြီးနောက် သင့်တွင်မည်သည့်အပြောင်းအလဲများ ရှိပါသလဲ။*  *သုတေသနသည်သင်နှင့်သင့်မိသားစု အပေါ် မည်သည့်သက်ရောက် မှု များ ရှိပါသလဲ၊*  *သုတေသနပြုလုပ်သူများ ဘာလုပ် မည်ကို သင်စိုးရိမ်ပူပန်မှုရှိပါလား။ ဘာကြောင့်ရှိပါသလဲ/ဘာကြောင့် မရှိပါသလဲ။*  *သုတေသနတွင် ပါဝင်ခဲ့ခြင်း အတွက် သင်နောင်တ ရခဲ့ပါ သလား။* |
|  | ၁၁။ သုတေသနတွင် အသုံး ပြုသော HIV ပိုးစစ်ဆေး ခြင်း နည်းလမ်းသစ် သင်မည်သို့ ထင်ပါသလဲ။ | *ထိုစစ်ဆေးမှုအကြောင်းကို သင် ယခင်က ကြားဖူးပါသလား။*  *ထိုစစ်ဆေးမှုနှင့်ပတ်သက်သော မည်သည့်အကြောင်းအရာများကို သင့်အားပြောပြခဲ့ပါသလဲ။*  *နေ့ချင်းပြီးအဖြေရမည့်အကြောင်း သင့်အားပြောကြားခဲ့သည့်အခါ ထို စစ်ဆေးမှုအပေါ် သင်မည်သို့ ထင် မြင်ပါသလဲ။* |
| *HIV* နှင့်ပတ်သက်သောမေးခွန်းအချို့ ကျွန်ုပ်မေးပါမည်။ အချို့မေးခွန်းများသည် သင့်ကို စိတ်ကသိကအောက်ဖြစ်စေနိုင်ပါသည်။ သင်မဖြေလိုသော မေးခွန်းများကို ဖြေစရာမလိုဘဲ မေးခွန်းမေးမြန်းခြင်းကိုလည်း အချိန်မရွေးရပ်နိုင်ပါသည်။ | | |
| *ကလေး၏*HIV*စစ်ဆေးမှုအပေါ် နားလည်မှုနှင့်* ART ဆေး သောက်ရန်လိုအပ်ချက်နှင့် မည်သို့ဆက်စပ်ခြင်း | ၁၂။ သင်ပိုးရှိမှန်းသိသည့် အခါ (သို့) ဤဆေးခန်းသို့ ပထမ ဆုံးလာသည့်အခါ သင့်ကလေး ကို HIV*စစ်ရန် ဆရာဝန်များ/ သူနာပြုများ မှ သင့်အား ဘာပြောပါသ လဲ။* | *သင်မည်မျှကြာအောင်စောင့်ရမည် ဟု သူတို့ပြောပါသလား။*  *အဖြေစောင့်နေစဥ်အတွင်း သင့် ကလေးကို ပြုစုစောင့်ရှောက် ရန် အတွက် မည်သည်တို့ကို ပြောပြ ပါသလဲ။*  *သင့်ကလေးတွင်* HIV *ပိုးရှိပါက ဘာဖြစ်နိုင်သည်ကို ပြောပါသလဲ။*  *သင့်ကလေးမွေးပြီးနောက် သောက် ရမည့်ဆေးများနှင့်ပတ်သက်၍ မည်သည်တို့ကို ပြောပါသလဲ။* |
| ART ကာကွယ်ဆေး သောက် ခြင်း | ၁၃။ *သင့်ကလေးမွေးပြီး နောက်* HIVပိုးကာကွယ် ရန် *သောက်ရသော ဆေးများ နှင့် ပတ်သက်၍ ပြောပြပါ။* | *သင့်ကလေးမွေးပြီး မည်မျှ ကြာ သည့်အခါ ထိုဆေးများတိုက် ရန် ပေးပါသလဲ။*  *သင့်ကလေးအားဆေးတိုက်ရာတွင် အခြေအနေ မည်သို့ရှိပါသလဲ။*  *မည်သည့်အခက်အခဲများရှိပါသလဲ။*  *မည်သည့်ပံ့ပိုးပေးမှုများရခဲ့ပါသလဲ။*  *မည်သည့် စိုးရိမ်မှု၊ ပူပန်မှုများ ရှိခဲ့ပါသလဲ။* |
| *ကလေးအားစောလျင်စွာ* HIV *စစ်ဆေးမှု အတွေ့အကြုံ များ* | ၁၄။ သင့်ကလေးအား HIV စစ်ဆေးမှုပြုလုပ်သောနေ့ တွင် အခြေအနေ မည်သို့ ရှိပါသလဲ။ | *ထိုနေ့အကြောင်းပြောပြပါ။*  *သင့်တွင်စိုးရိမ်ပူပန်မှုများ ရှိခဲ့ပါ သလား။*  *သင့်အားလာခိုင်းသောနေ့တွင် သင်လာပါသလား။*  *ဆရာဝန် (သို့) သူနာပြုများကို တွေ့ ရန် သင်မည်မျှကြာအောင် စောင့် ရပါသလဲ။*  *သင်စောင့်နေစဥ်အတွင်း ဘာလုပ် ပါသလဲ။* |
|  | ၁၅။ သင့်ကလေးကို သွေး စစ်ဆေးရန် ခေါ်ဆောင် လာရသည့် အကြောင်းရင်း များကို ပြောပြပါ။ | *သင့်ကလေးကို ဆေးခန်းသို့ ခေါ်လာ ရာတွင် မည်သည့်အရာများက လွယ်ကူ စေခဲ့ပါသလဲ။*  *မည်သည့်အရာများက ခက်ခဲစေ ခဲ့ပါသလဲ။* |
|  | ၁၆။ သင့်ကလေး၏ သွေး အဖြေစောင့်ရာတွင် အခြေ အနေ မည်သို့ ရှိခဲ့သည်ကို ပြောပြပါ။ | *သွေးအဖြေရရန် မည်မျှကြာအောင် စောင့်ရပါသလဲ။*  *စောင့်နေစဥ်အတွင်းသင်ဘာလုပ် ပါသလဲ။* |
|  | ၁၇။ သင့်ကလေး၏သွေး အဖြေကို ဆရာဝန်/သူနာ ပြုဆရာမ မှပြောသည့် အကြောင်းကို ပြောပြပါ။ | *သင့်အား မည်သို့ပြောပြပါသလဲ။*  *ဘာတွေပြောပါသလဲ။*  *သင့်အားမည်သည့်ညွှန်ကြားချက်များ ပေးပါသလဲ။*  *သွေးအဖြေရသည့်အတွက် သင်မည် သို့ဖြစ်ပါသလဲ။ မည်သို့ခံစားရပါသ လဲ။*  *သင့်တွင်မည်သည့်စိုးရိမ်ပူပန်မှုများ ရှိပါသလဲ။*  *Did you tell anyone? Who did you tell?*  *တစ်စုံတစ်ယောက်ကိုပြောပြခဲ့ ပါသလား။ မည်သူ့ကို သင်ပြောပြ ပါသလဲ။* |
|  | ၁၈။ **အဖြေ တွင်ပိုးတွေ့ ပါက၊** ဆရာဝန်များ/ သူနာပြုများမှ သင့်ကလေး တွင် HIVရှိကြောင်းကို ဘာတွေပြောပြပါသလဲ။ | *HIV ပိုးအတွက်ကုသမှုအကြောင်း ဘာတွေပြောပြပါသလဲ။*  *သင့်ကလေးကုသမှုခံယူနေပါသလား။ သွေးအဖြေရပြီး မည်မျှ အကြာတွင် ကုသမှုရရှိခဲ့ပါသလဲ။*  *ကုသမှုအတွက်မည်မျှကြာအောင် စောင့်ခဲ့ရပါသလဲ။ နောက်ထပ် ရက်ချိန်းအတွက် သင်ပြန်လာခဲ့ ရပါ သလား။*  *သင့်ကလေးကုသမှုစတင်ခံယူ သည့် အခါ အခြေအနေမည်သို့ရှိပါသလဲ။*  *မည်သည့်အခက်အခဲများ ရှိပါသလဲ။*  *သင့်ကလေးအတွက် လုံလောက် သောကုသမှု ရရှိနေပါသလား။*  *ကလေးတွင်HIV ပိုးရှိကြောင်း သိသွားပြီးနောက် ကလေးကို ပြုစု စောင့်ရှောက်မှုများ မည်သို့ပြောင်း လဲသွားပါသလဲ။* |
|  | ၁၉။ **အဖြေ တွင်ပိုးမတွေ့ ပါက၊** ဆရာဝန်များ/ သူနာပြုများမှ သင့်ကလေး သွေးအဖြေအကြောင်းကို ဘာတွေ ပြောပြပါသလဲ။ | *ထပ်မံပြုလုပ်ရမည့်သွေးစစ်ဆေး မှုအကြောင်းကို သင့်အား ဘာတွေ ပြောပြပါသလဲ။*  *ကလေးတွင်HIV ပိုး မရှိကြောင်း သိသွားပြီးနောက် ကလေးကို ပြုစု စောင့်ရှောက်မှုများ မည်သို့ပြောင်း လဲသွားပါသလဲ။*  *သင့်ကလေးတွင်ပိုးမတွေ့ကြောင်း နှင့် ၆ လ အထိမိခင်နို့တိုက်နိုင် ကြောင်း သင့်အား ပြောသည့်အခါ သင်မည်သို့ ထင်ပါသလဲ။* |
| *သင့်ကလေး၏HIV စစ်ဆေးမှုသည် တစ်နာရီ မိနစ်၃၀ ခန့် ကြာပါသည်။ ဓါတ်ခွဲခန်းကြီးများတွင် ပုံမှန် စစ်ဆေးသည့် နည်းလမ်းသည် သွေးအဖြေရရန်တစ်လမှ နှစ်လကြားကြာပါသည်။* | | |
| နေ့ချင်းပြီးသွေးအဖြေကို လက်ခံနိုင်မှု | ၂၀။ သင့်ကလေးသွေးအဖြေ ကို နေ့ချင်းပြီး ရသည့် အတွက် သင်မည်သို့ ထင်ပါသလဲ။ | *သင့်ကလေးသွေးအဖြေ ဘာဖြစ် မည်ဆိုသည်ကို လက်ခံရန် သင့် တွင် ပြင်ဆင်ချိန်ရှိခဲ့ပါသလား။*  *သွေးစစ်သည့်နေ့တွင်ပင် သွေး အဖြေ ရသည့်အပေါ် မည်သည့် ကောင်းကျိုး နှင့် ဆိုးကျိုးများ ရှိမည်ဟု သင်ထင်ပါသလဲ။*  *သင့်တွင်အခြားကလေးများရှိပါက ၄င်းတို့၏စစ်ဆေးမှုနှင့် ယခု သုတေ သနတွင် စစ်ဆေးမှုတို့မည်သို့ ကွာ ခြားပါသလဲ။*  *မတူညီသောနည်းလမ်းများ၏ ကောင်းကျိုး နှင့် ဆိုးကျိုးကို ပြောပြ ပါ။*  *သင့်တွင် ရွေးချယ်စရာရှိပါက မည်သည့်စစ်ဆေးမှုကို ရွေးချယ် မည်နည်း။ တစ် နာရီ နှင့်မိနစ် ၃၀ ကြာသော စစ်ဆေးမှု ကို ပြုလုပ် မည်လား (သို့) လအနည်းငယ် ကြာသော စစ်ဆေး မှုကို ပြုလုပ် မည်လား။*  *ဘာကြောင့်ထိုသို့ရွေးချယ်ပါသလဲ။* |
| နေ့ချင်းပြီး စစ်ဆေးမှုအတွက် တိကျမှန်ကန်မှု (ဓါတ်ခွဲခန်းကြီး များတွင် ပြုလုပ်သော နည်းလမ်းများ နှင့် နှိုင်းယှဥ်ပါက) | ၂၁။ ကလေးများတွင် HIV စစ်ဆေးလေ့ရှိသောနည်း လမ်းများအကြောင်း ကို ပြောပြပါ။ | *သွေးနမူနာများကို မည်သူက စစ်ပါသလဲ။*  *မည်သည့်နေရာတွင် စစ်ပါသလဲ။*  *သွေးအဖြေရရန် မည်မျှကြာပါသလဲ။* |
|  | ၂၂။ ပုံမှန်အားဖြင့် ဆေးခန်း မှ သွေးနမူနာများကို ဓါတ် ခွဲခန်းသို့ပို့၍ HIV စစ်ဆေး ပါသည်။ ဆေးခန်းတွင် စစ် ဆေးခြင်း နှင့် ဓါတ်ခွဲခန်း သို့ ပို့၍စစ်ဆေးခြင်း တို့ကို ယှဥ် လျှင် သင်မည်သို့ထင်ပါ သလဲ။ | *ဓါတ်ခွဲခန်းတွင်စစ်ဆေးခြင်း ထက် ဆေးခန်းတွင်စစ်ဆေးမှုအပေါ် မည်သို့ထင်ပါသလဲ။*  *ဤနေရာတွင်စစ်ဆေးခြင်း နှင့် ဓါတ်ခွဲခန်း တွင် စစ်ဆေးခြင်းတို့ ယုံကြည်စိတ်ချရမှုတွင် မည်သို့ကွာ ခြားသည်ဟု ထင်ပါ သလဲ။*  *သုတေသနတွင် စစ်ဆေးမှု အဖြေ များသည် မည်မျှ ယုံကြည်စိတ်ချ နိုင်သည်ဟု သင်ထင်ပါသလဲ။*  *သွေးအဖြေကို သင်ယုံကြည်ပါသ လား။*  *သူတို့ကို မေးခွန်းများပြန်မေးပါ သလား။ ဘာကြောင့်မေးပါသ လဲ။* |
| အဆုံးသတ်မေးခွန်း | ၂၃။ HIVပိုးရှိသော အခြား မိခင်များ၊ မိဘများ ကို ဤစစ်ဆေးမှု၊ ကောင်းကျိုး များ၊ အဆင် မပြေမှုများ အကြောင်းကို သင် မည်သို့ ပြောပြချင်ပါသလဲ။ | *ဤစစ်ဆေးမှု၏ ကောင်းကျိုးများ ကို ပြောပြပါ။*  *အဆင်မပြေမှုအခက်အခဲများကို ပြောပြပါ။*  *ဆေးခန်းတွင်HIVပိုးစစ်ဆေးမှုကို မတူကွဲပြားစွာ ပြုလုပ်ချင်ပါသလား။ မည်သို့ပြောင်းလဲပြုလုပ်ချင်ပါသလဲ။* |
