# Supplementary material for: Caregiver experience and perceived acceptability of a novel near point-of-care early infant HIV diagnostic test among caregivers enrolled in the PMTCT program, Myanmar: A qualitative study
Source: PLoS One. 2020 Oct 30;15(10):e0241245. doi: 10.1371/journal.pone.0241245 (PMC7598472; doi:10.1371/journal.pone.0241245)
Supplement: Attachment — Submitted filename: Reviewers Comment _PMTCT.docx [file pone.0241245.s004.docx]

General comment

The topic is very important as the new POC EID test obviously lessen the waiting time of the caregivers to hear their infant's test result. However, this study has a methodological and coherence problem. First, the type of study should be clear. If this study is a comparative study, then there should be two distinct groups and measurement variables need to be presented comparatively to each group. For example, under the result section(L64-65), it says “the acceptability of the Xpert testing process was high among the caregivers …” but is not clear which group has high accessibility. I understand, there was a randomized control trial study. If you think, the parent study is helpful for the current study, the methodology of the main study should explicitly and separately explain. Similarly, the methods for the current qualitative study must be explained. Confusions with the current and previous studies must be cleared.

**Title** – The title doesn’t go with study objectives and methodology. The title may be revised as “assessment of caregiver experience and perceived acceptability of point-of-care among caregivers of HIV-exposed infants enrolled in the PMTCT program, Myanmar: A qualitative study/ a comparative study?

**Abstract** – methodology is not explicitly explained. Is it a comparative study? how were participants selected? How many participants were from the Xpert and standard of care tests?

**Introduction -**

**Methods -**

**L152-153 –** Shows this study has control and intervention phases. It seems a time series quasi-experimental study but there is no further evidence in your document to say it is an interventional study.

**L179-180 -** It indicates participants of the intervention phase were eligible for the current study if they have received both Xpert and the standard of care. what about at the control phase? Did you use the same participants in the control phase and intervention phase or different participants?

**L170-173** – Two things should be clear. If this study is comparative - there must be two groups or one group before and after the intervention. If you are asking participants to compare two things, it is just a perception assessment. It is difficult to evaluate the outcome of the intervention( POC EID in this case). Such kind perception and acceptability assessments are helpful for feasibility assessment before the implementation of the new program.

**Discussion -**

**L501-503 -**  Do you think lost from follow up is a problem in Myanmar? PMTCT lost a follow-up rate of 0.7% and 1% for mothers and infants, respectively are very low**.** So, I don’t see the importance of the POC EID test to reduce PMTCT lost follow-up rate

**L528-529** – stigma, and discrimination against HIV infection were found to be a problem in the study area. On the other hand, accessibility issues, transportation costs, and work absenteeism for appointment were **(L495-499)** among the reasons for lost follow-up. In a community where stigma a big problem, patients prefer to follow in clinics far from their residence. How do reconciled these two contradicting factors?

**Limitation**

L587-593, As a limitation it is stated that *more than half of the caregivers were mothers who had no previous experience with EID testing.* Against this, under the method section**(L179-180),** “*participants were eligible only if they had undergone EID and received Xpert and the standard of care results.”* Therefore, according to the eligibility criteria, the above statement couldn’t be a limitation. Please justify it.

**Conclusion**

L597 - I think an exposed infant caregiver is preferable instead of HIV positive mothers. the term 'caregiver' is used most often in this document including in the title.
