# Supplementary material for: Caregiver experience and perceived acceptability of a novel near point-of-care early infant HIV diagnostic test among caregivers enrolled in the PMTCT program, Myanmar: A qualitative study
Source: PLoS One. 2020 Oct 30;15(10):e0241245. doi: 10.1371/journal.pone.0241245 (PMC7598472; doi:10.1371/journal.pone.0241245)
Supplement: Attachment — Submitted filename: Response to reviewers.docx [file pone.0241245.s005.docx]

**Response to reviewers**

| **Comments** | **Responses** |
| --- | --- |
| Please include additional information regarding the interview guide used in the study and ensure that you have provided sufficient details that others could replicate the analyses. For instance, if you developed a guide as part of this study and it is not under a copyright more restrictive than CC-BY, please include a copy, in both the original language and English, as Supporting Information. In addition, please provide further details of the pilot testing of this tool. | The manuscript is revised according to the suggestion. Please see L201-205 of the revised manuscript with track changes.  Original language (Myanmar) and English versions of the interview guide are submitted as Supporting Information. |
| Please ensure the clinical trial this is related to is written correctly as “Australian and New Zealand Clinical Trials Registry, number 12616000734460” could not be found. | This has been revised at L52-54, L144-146 of the revised manuscript with track changes. |
| We note that you have indicated that data from this study are available upon request. PLOS only allows data to be available upon request if there are legal or ethical restrictions on sharing data publicly. | The justification for data unavailability is provided in the cover letter. |
| One of the noted authors is a group or consortium [AAMI study group]. In addition to naming the author group, please list the individual authors and affiliations within this group in the acknowledgments section of your manuscript. Please also indicate clearly a lead author for this group along with a contact email address. | Group authors are listed in the acknowledgement section (L633-644 of the revised manuscript with track changes). |
| If you think, the parent study is helpful for the current study, the methodology of the main study should explicitly and separately explain. Similarly, the methods for the current qualitative study must be explained. Confusions with the current and previous studies must be cleared. | The methodology has been revised and detailed in both abstract and the main manuscript. |
| **Title** – The title doesn’t go with study objectives and methodology. The title may be revised as “assessment of caregiver experience and perceived acceptability of point-of-care among caregivers of HIV-exposed infants enrolled in the PMTCT program, Myanmar: A qualitative study/ a comparative study? | Title has been changed as advised. |
| **Abstract** – methodology is not explicitly explained. Is it a comparative study? how were participants selected? How many participants were from the Xpert and standard of care tests? | More information about the methods of the study is provided in L 52-56 of the revised manuscript with track changes. |
| **L152-153 –** Shows this study has control and intervention phases. It seems a time series quasi-experimental study but there is no further evidence in your document to say it is an interventional study. | The study setting and study design has been revised to be clearer between the main study and the qualitative sub-study. (L144-146, L160, 161, 175,184 of the revised manuscript with track changes) |
| **L179-180 -** It indicates participants of the intervention phase were eligible for the current study if they have received both Xpert and the standard of care. what about at the control phase? Did you use the same participants in the control phase and intervention phase or different participants? | We interviewed only the intervention phase participants to understand their Xpert testing experience compared to standard of care testing. Please see L191 of the revised manuscript with track changes. |
| **L170-173** – Two things should be clear. If this study is comparative - there must be two groups or one group before and after the intervention. If you are asking participants to compare two things, it is just a perception assessment. It is difficult to evaluate the outcome of the intervention( POC EID in this case). Such kind perception and acceptability assessments are helpful for feasibility assessment before the implementation of the new program. | L158-161 of the revised manuscript with track changes described the design of the main study.  L166-172 of the revised manuscript with track changes detailed the control phase participants and the intervention phase participants.  L190-195 of the revised manuscript with track changes explained the eligible participants for qualitative sub-study. |
| **L501-503 -**  Do you think lost from follow up is a problem in Myanmar? PMTCT lost a follow-up rate of 0.7% and 1% for mothers and infants, respectively are very low**.** So, I don’t see the importance of the POC EID test to reduce PMTCT lost follow-up rate | Lost to follow-up is evident in the previous study. Seven per 1000 person-years is the incidence rate taking into account of the different time the mothers became lost to follow-up. I have changed it with the proportions of mothers and infants who became lost to follow-up at L519-521 of the revised manuscript with track changes. |
| **L528-529** – stigma, and discrimination against HIV infection were found to be a problem in the study area. On the other hand, accessibility issues, transportation costs, and work absenteeism for appointment were **(L495-499)** among the reasons for lost follow-up. In a community where stigma a big problem, patients prefer to follow in clinics far from their residence. How do reconciled these two contradicting factors? | Please see L553-555 of the revised manuscript with track changes that addressed this concern. |
| L587-593, As a limitation it is stated that *more than half of the caregivers were mothers who had no previous experience with EID testing.* Against this, under the method section**(L179-180),** “*participants were eligible only if they had undergone EID and received Xpert and the standard of care results.”* Therefore, according to the eligibility criteria, the above statement couldn’t be a limitation. Please justify it. | This has been revised. (L608 of the revised manuscript with track changes) |
| L597 - I think an exposed infant caregiver is preferable instead of HIV positive mothers. the term 'caregiver' is used most often in this document including in the title. | This has been revised according to the suggestion. (L616 of the revised manuscript with track changes). |
